# Age and Sex Effects on the Active Stiffness of Vastus Intermedius under Isometric Contraction

**DOI:** 10.1155/2017/9469548

**Published:** 2017-04-03

**Authors:** Cong-Zhi Wang, Jing-Yi Guo, Tian-Jie Li, Yongjin Zhou, Wenxiu Shi, Yong-Ping Zheng

**Affiliations:** ^1^Paul C. Lauterbur Research Center for Biomedical Imaging, Institute of Biomedical and Health Engineering, Shenzhen Institutes of Advanced Technology, Chinese Academy of Sciences, Shenzhen, China; ^2^Interdisciplinary Division of Biomedical Engineering, the Hong Kong Polytechnic University, Hong Kong; ^3^School of Biomedical Engineering, Shenzhen University, Shenzhen, China

## Abstract

Previously, a novel technique was proposed to quantify the relationship between the muscle stiffness and its nonfatigue contraction intensity. The method extended the measured range of isometric contraction to 100% maximum voluntary contraction (MVC) using an ultrasonic shear wave measurement setup. Yet, it has not been revealed how this relationship could be affected by factors like age or sex. To clarify these questions, vastus intermedius (VI) stiffness of 40 healthy subjects was assessed under 11 step levels of isometric contraction. The subjects were divided into four groups: young males, young females, elderly males, and elderly females (*n* = 10 for each). In a relaxed state, no significant difference was observed between the male and female subjects (*p* = 0.156) nor between the young and elderly subjects (*p* = 0.221). However, when performing isometric contraction, the VI stiffness of males was found to be significantly higher than that of females at the same level (*p* < 0.001), and that of the young was higher than the elderly (*p* < 0.001). Meanwhile, for two knee joint angles used, the stiffness measured at a 90° knee joint angle was always significantly larger than that measured at 60° (*p* < 0.001). Recognizing the active muscle stiffness of VI contributes to body stability, and these results may provide insight into the age and sex bias in musculoskeletal studies, such as those on fall risks.

## 1. Introduction

Skeletal muscle is the largest tissue within the fat-free human body mass. Its primary function, force generation, is based on its ability to contract voluntary. The neuromuscular activity during muscle contraction has been studied in depth by means of electromyography (EMG) and mechanomyography (MMG) signals (e.g., [1, 2]). Similar studies have also been performed by morphological parameters, such as muscle thickness, pennation angle, fascicle length, and cross-sectional area (CSA), which can be estimated in vivo from medical images [3, 4]. In particular, the age and sex effects on these neuromuscular features and morphology characteristics have become the focus of research. The degenerative loss of skeletal muscle mass and strength in elderly humans have been confirmed and were found to be associated with the increased susceptibility to fall and a decreasing mobility [5–8]. The higher risk of injury among females on their joints was also found to be related to the sex differences of the skeletal muscle characteristics [9–11]. Furthermore, for voluntary muscle contraction under different joint angles, EMG activity and some morphology characteristics have been proved to also change [12–15]. In addition to neuromuscular features and morphological characteristics, recently the mechanical properties of skeletal muscle have attracted more attention. Skeletal muscle is typically composed of contractile (myosin) and passive elastic (actin and connective tissues) components. Stiffness, which is observed in both active and passive muscle behaviours, has been shown to contribute significantly to muscle efficiency [16]. Passive stiffness of muscle is important for the control of movement because it determines the muscle resistance to external perturbations, while active muscle stiffness plays a key role in both force generation and body movements. It is well-known from simple palpation that muscle stiffness increases during voluntary contraction, and quantifying muscle stiffness under different voluntary contraction levels can help us better understand the muscle recruitment strategies. Subsequently, a number of methods have been developed for noninvasive muscle stiffness assessment, such as the indentation assessment method [2], sonoelastography [17], transient elastography [18], supersonic shear imaging (SSI) [19, 20], shear wave dispersion ultrasound vibrometry (SDUV) [21], and magnetic resonance elastography (MRE) [22–24]. It has been reported that different muscles exhibited different stiffness in a relaxed state [23], and it has been also found that the stiffness of skeletal muscle is positively correlated to its nonfatigue contraction intensity within a small range of isometric contraction levels, that is, from 0% to 20–60% maximum voluntary contraction (MVC) levels [18, 20, 22, 24]. However, due to the limitation of the measurement range, this conclusion has rarely been verified over the entire range of isometric contraction, that is, from 0% to 100% MVC. In our previous study [25, 26], a vibroultrasound setup has been reported to assess the shear modulus of skeletal muscle along the direction of muscle action. The positive correlation between the shear modulus of vastus intermedius (VI) and the relative isometric contraction level (% MVC) of the knee extensor has been verified over the entire range of isometric contraction on young and elderly healthy females subjects. However, the effects of different age ranges and sex on the relationship between muscle stiffness and isometric contraction levels have not been systematically studied, although some related studies have been performed separately [26–29], but none over the entire range of MVC. To clarify these questions, vastus intermedius, one of the quadriceps femoris muscles, was selected as the target of this study. Quadriceps femoris is a muscle group including four powerful extensors of knee joint on the front of the thigh. VI lies between vastus lateralis (VL) and vastus medialis (VM), right under the rectus femoris (RF) and above the femur. They are the strongest and leanest muscles of the human body and are crucial in walking, running, jumping, and squatting [30]. Previously, several studies have been performed to investigate the structure, function, and characteristics of quadriceps femoris muscles, including their mechanical properties [5, 22, 23, 31]. In addition, the sex difference between adult men and women [32] and the age difference between young and elderly adults [5] of the morphological parameters have also been compared. In the present study, VI stiffness was assessed on the subjects from four different subject groups: young males, young females, elderly males, and elderly females (*n* = 10 for each group), and the experiments were repeated at two different knee joint angles, 90° and 60° (0° corresponds to full extension for all joint angles in this paper). The age and sex effects on VI stiffness over the entire range of step isometric contraction were systematically analyzed and discussed. It is believed that these results could provide new information regarding the phenomenon, such as the higher risk of ACL (anterior cruciate ligament), cartilage injury, or fall among females than males, and facilitate investigations on the process of muscle ageing and the probability of its rehabilitation.

## 2. Materials and Methods

### 2.1. Ethics Statement

In this study, human subject ethical approval was obtained from the Human Ethics Committee of the Hong Kong Polytechnic University, and the experimental protocol was explained to all of the subjects and they were asked to sign the informed consent form prior to the experiment.

### 2.2. Subjects Selection

Forty healthy subjects volunteered to participate in the experiments and were divided into four groups: young males, young females, elderly males, and elderly females (Table 1). They were asked not to participate in any strength or flexibility training one day before the experiment.

The experimental setup was almost the same as that described in our previous study [26].

**Figure 1:**
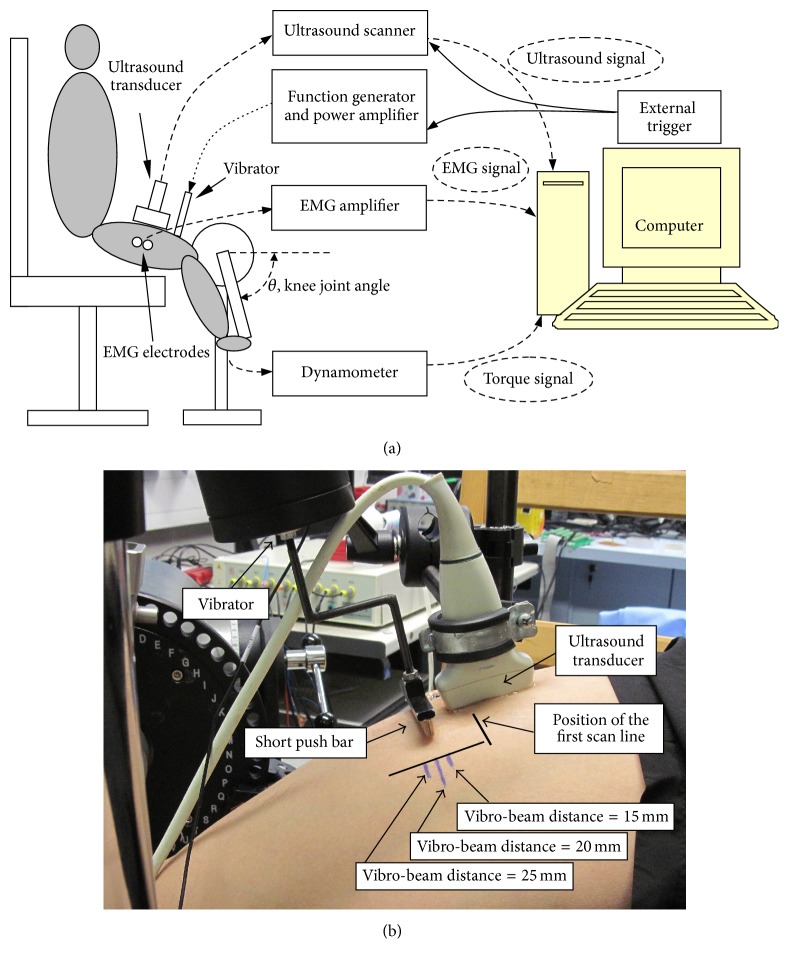
Illustration (a) and photo (b) of the experiment setup for human subjects.

The vibroultrasound system consists of a mechanical vibrator, a programmable ultrasound scanner, and a custom-made program for radiofrequency data acquisition. An electromagnetic vibrator (minishaker type 4810, Brüel and Kjær, Nærum, Denmark), which was driven by a power amplifier and controlled by a function generator, was used to induce transient low-frequency shear waves (monochromatic sinusoidal pulse). An ultrasound linear array probe was placed along the muscle action direction, so that the tissue movements in response to external mechanical vibration can be monitored by two separated ultrasound scan lines (to spatially sample the induced shear waves), as shown in Figures 1(a) and 1(b) [26]. The distance between these two lines was Δ*r* (15 mm in this study) and the time delay between the two positions was Δ*t*.

Then the shear wave velocity *c*_*s*_ could be calculated by

(1) $$\begin{array}{c} c_{s}=\frac{\Delta r}{\Delta t}. \end{array}$$

The shear modulus *μ* of the measured muscle can then be calculated via the following equation:

(2) $$\begin{array}{c} \mu =\rho {c_{s}}^{2}, \end{array}$$

where *ρ* was the mass density of the muscle tissue, using a reported approximate value as 1000 kg/m^3^. The equation and the parameter value used have been proved to be promising for estimating the shear modulus of skeletal muscle in many previous studies [22–24, 26, 33]. The data acquisition part was developed based on a commercial ultrasound scanner SonixRP (Ultrasonix Medical Corp. Vancouver, Canada) with a 5–14 MHz linear array probe. B-mode images were first acquired using a predefined penetration depth (65 mm in this study) to help position the probe. When triggered by an external signal of starting vibration, B-mode imaging was stopped and two scan lines were repeated with a high frame rate (4.6 kHz in this study). The tissue movements were then estimated by an improved cross-correlation algorithm and the shear wave velocity and shear modulus were calculated. Isometric torque generated by the knee extensors was assessed using a HUMAC NORM rehabilitation system (Computer Sports Medicine, Inc., Stoughton, MA, USA). The machine was set to a knee joint isolated movement pattern and isometric resistance mode, under which the knee joint angle can be set and fixed. The EMG signals were also captured from the surface of VL muscle, but the results were not included in this paper. The whole experimental setup for human subjects is illustrated in Figure 1.

### 2.3. Experimental Protocol

At first, the ultrasound probe was placed on the middle part of the RF muscle belly right above the VI and femur with the guidance of B-mode images. The distance between the probe and vibrator was set to be approximately 10 mm (i.e., the distance between the short push bar and the proximal scan line was about 20 mm). The MVC torque was first assessed as the highest torque value of subjects produced from three successive isometric contractions, when subjects were asked to put forth all of their strength to extend their knee joints. Next, the muscle stiffness was measured three times in a relaxed state. Then the subject was asked to maintain isometric contraction at different levels, from 10% to 100% MVC, with an increase of 10% MVC for each step. At each level, assessments were performed for three times with about a 1 min interval for a rest to avoid fatigue. For each trial, the subject was asked to maintain the isometric contraction for approximately 4 s. Experiments were performed at two different knee joint angles, 90° and 60°.

### 2.4. Data Analysis

Part of the data, measured on the young and elderly female subjects at a 60° knee joint angle, has been presented and analyzed in our previous study [26]. A more detailed comparison was performed in this paper on the data of all four groups of subjects. A total of 2640 (4 [groups: young males, young females, elderly males, and elderly females] × 10 [subjects per group] × 11 [contraction levels: 0%–100% MVC] × 2 [knee joint angles] × 3 [measured three times]) shear modulus assessments were performed by the same investigator. To study the influences of the interested factors, including age and sex, on the muscle stiffness in a relaxed state and different isometric contraction levels, four-way repeated measure analyses of variance (ANOVA) (age [young and elderly] × sex [male and female] × knee joint angles [90° and 60°] ×  % MVC [0%–100%, 11 levels]) were used to analyze the shear modulus of the VI. Specially, the comparison of the VI shear modulus measured in a relaxed state (0% MVC) was first performed separately using a three-way ANOVA method.

Two methods were used to exhibit the different contraction intensities; one was to use the relative muscle contraction level (% MVC), and the other one was to use the absolute torque of the knee joint extensors. These two methods focused on different aspects. Using the former aspect, muscle stiffness was studied at different relative levels of the maximal muscle contraction capability for each person, considering the individual muscle strength difference. On the other hand, using the later aspect, muscle stiffness was generally evaluated corresponding to the absolute torque and it is meaningful in a specific group of people. The results of both methods could help us to further understand the muscle function and recruitment strategies from different perspectives, that is, from the perspective of the changes of its mechanical properties over the entire range of step isometric contraction, besides the perspective of neuroelectric activity, which is conventionally measured by electromyography (EMG). In our study, to determine the relationship between the VI stiffness and relative isometric contraction level (% MVC), polynomial regression analyses by linear, quadratic, and cubic models were performed for each individual, and the coefficients of determination (*R*^2^) values of these models were compared using one-way ANOVA to find the best model. The mean shear modulus values across the ten subjects in each group then were fitted with the relative isometric contraction level (% MVC) using a quadratic regression model. From another perspective, to study the relationship between the VI shear modulus and the corresponding absolute torque, quadratic regression was performed for each individual subject and the 10 sets of polynomial coefficients in each group were averaged to represent the trend of this relationship. The achieved quadratic curve was plotted in a range from 0 N·m to the mean MVC torque value in each group. All the data were analyzed using SPSS (SPSS Inc., Chicago, IL, USA). Statistical significance was set at the 5% probability level.

## 3. Results

### 3.1. Results of Multiway ANOVA Analysis

Firstly, the mean MVC torques of the knee extensors in each group, and for different knee joint angles, are listed in Table 2. The results of the three-way ANOVA showed that the main effects of age (*p* = 0.01), sex (*p* < 0.001), and knee joint angle (*p* = 0.001) factors were all significant for the mean MVC torque. However, all of the two-way interaction effects were not significant (all *p* > 0.1). According to the estimated marginal mean values provided by the ANOVA method (the marginal means for one factor are the means for that factor averaged across all levels of the other factors), the conclusion was that the mean MVC torque for males was larger than that for females; the values from young subjects were larger than those from elderly subjects; the values measured at the 90° knee joint angle were larger than those measured at 60°.

Comparison of the VI shear modulus in a relaxed state (0% MVC) was performed first. The means of VI shear modulus from each group are shown in Table 3. Results of the multiway ANOVA showed that there was no significant main effect for sex (*p* = 0.156) and age (*p* = 0.221) factors, and an interactive effect between each of the two factors (all *p* > 0.05) on the VI shear modulus was measured in a relaxed state. However, there was a significant main effect for the knee joint angle factor (*p* = 0.001) on the VI shear modulus. The estimated marginal mean value of the VI shear modulus measured at 90° knee joint angle (16.2 ± 8.1 kPa) was larger than the mean value at 60° knee joint angle (11.1 ± 4.4 kPa) in this state.

**Figure 2:**
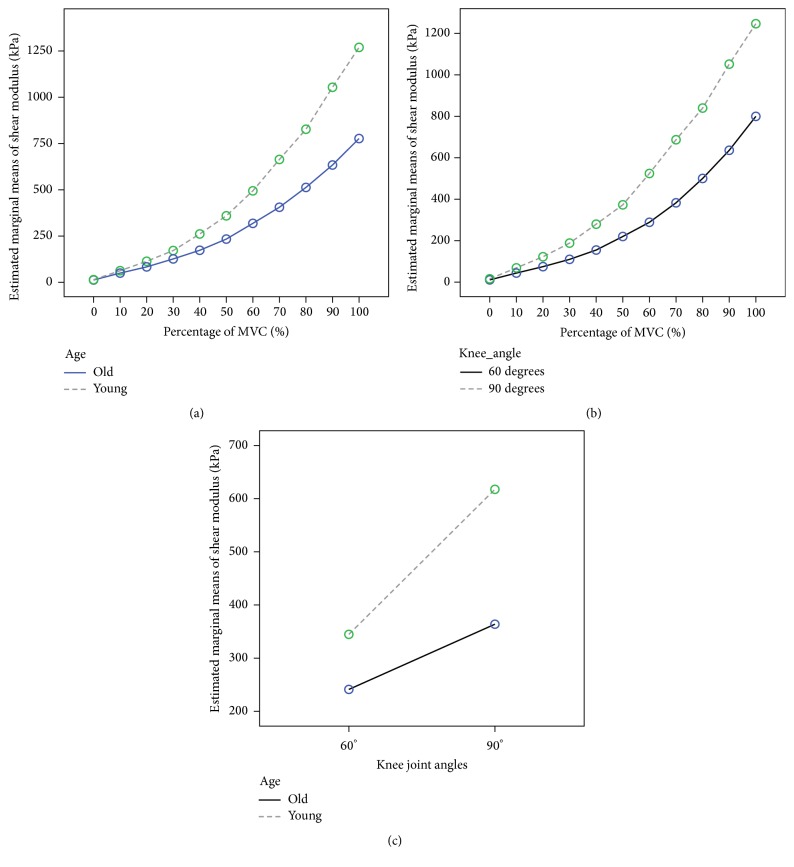
Estimated marginal means of VI shear modulus plotted for illustrating the significant two-way interaction effects for (a) age versus % MVC. (b) Angles versus % MVC. (c) Age versus joint angles.

For the VI shear modulus measured under different step isometric contraction levels, there were no significant four- or three-way interaction effects among the four factors (sex × age ×  % MVC × knee joint angles, all *p* > 0.05). All of the main effects of the single factor on the VI shear modulus were significant. The estimated marginal mean value of males was larger than that of females (*p* < 0.001). The mean value of young subjects was larger than that of elderly participants (*p* < 0.001), and the mean value measured at a 90° knee joint angle was larger than that measured at 60° (*p* < 0.001). In addition, the results of the post hoc Bonferroni test for the VI shear modulus measured at different percentages of MVC levels are shown in Table 4, which shows the detailed effects of the relative isometric contraction level on VI stiffness. Furthermore, all of the two-way interactions, including the sex factor, were not significant (all *p* > 0.1) and all other two-way interaction effects, that is, age and % MVC, joint angles and % MVC, and age and joint angles, were significant (all *p* < 0.001). For age and % MVC interaction, with an increase in the % MVC, differences between the VI shear modulus of young subjects and that of elderly subjects also increased. A similar relationship was also found for joint angles and % MVC; with an increase in the % MVC, differences between the VI shear modulus measured at a 90° knee joint angle and that at 60° also increased. For age and joint angles, the differences of VI shear modulus between the young and elderly subjects were larger at a 90° knee joint angle than at 60°. The detailed relationships are shown in Figure 2 by plotting the estimated marginal means of the VI shear modulus together.

### 3.2. Analysis Based on Relative Isometric Contraction Level (% MVC)

**Figure 3:**
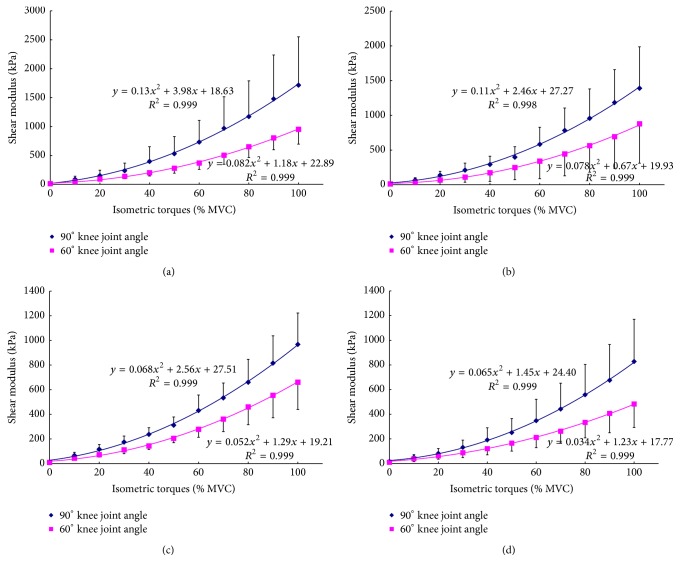
The mean VI shear modulus at different relative isometric contraction levels (% MVC) and different knee joint angles (90° and 60°) with the corresponding quadratic regression fitting curves for (a) young male subjects; (b) young female subjects; (c) elderly male subjects; and (d) elderly female subjects.

Polynomial regression analyses by linear, quadratic, and cubic models were performed on the relationship between VI shear modulus and relative isometric contraction level (% MVC) for each individual and the mean  *R*^2^  of three models were 0.937 ± 0.034 (linear), 0.992 ± 0.007 (quadratic), and 0.995 ± 0.004 (cubic), respectively. The results of the one-way ANOVA indicated that there was no significant difference between the *R*^2^ of the quadratic model and cubic model (*p* = 0.390). However, significant differences were found between the *R*^2^ of the linear model and the other two models (all *p* < 0.001). Thus, the quadratic model was selected to examine the relationship of the VI shear modulus and relative isometric contraction level. The mean shear modulus across the ten subjects in each group at the same knee joint angle was fitted using a quadratic regression model with the relative isometric contraction level, as shown in Figure 3. Pearson's correlation coefficients (CC) between VI shear modulus and % MVC level in each group and knee joint angle are listed in Table 5, which were all larger than 0.96. This indicated that the VI stiffness along the muscle action direction was positively correlated to the relative muscle activity intensity level of the knee extensors over the entire range of step isometric contraction. The plots also indicated that the VI shear modulus measured at a 90° knee joint angle was larger than the corresponding value measured at a 60° knee joint angle at almost each % MVC level in each group of subjects, which was in agreement with the result of the multiway ANOVA analysis.

**Figure 4:**
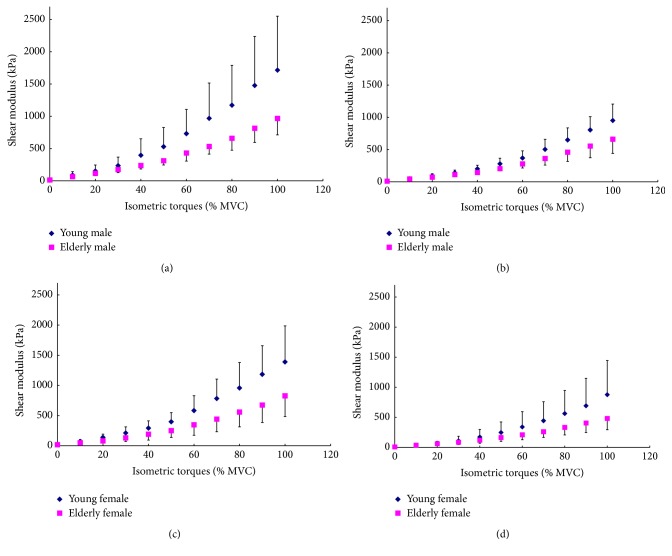
The age effects on the mean VI shear modulus at different relative isometric contraction levels (% MVC) between the subjects with same gender. Comparisons were made between young and elderly male subjects at (a) 90° and (b) 60° knee joint angles and between young and elderly female subjects at (c) 90° and (d) 60° knee joint angles.

**Figure 5:**
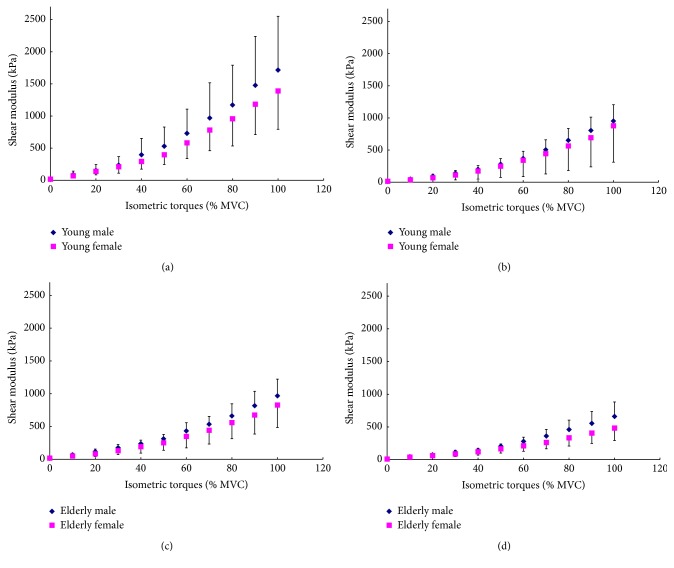
The gender effects on the mean VI shear modulus at different relative isometric contraction levels (% MVC) between subjects with similar age range. Comparisons were made between young male and female subjects at (a) 90° and (b) 60° knee joint angles, and between elderly male and female subjects at (c) 90° and (d) 60° knee joint angles.

The results between young and elderly groups with the same sex at the same knee joint angle were compared, as shown in Figure 4. It was observed that, for both sexes and knee joint angles, the VI shear moduli of young subjects were larger than those of elderly participants, especially at the relatively higher % MVC level. The results were also compared between the two sexes with a similar age range at the same knee joint angle, as shown in Figure 5. The comparison showed that for both age ranges and knee joint angles, the VI shear moduli of males were larger than those of females, especially at the relatively higher % MVC level. However, at the corresponding relative muscle contraction level, the sex difference seemed to be smaller than the age difference. These results were both in agreement with those obtained using multiway ANOVA that the main effects of age and sex factors were significant.

### 3.3. Analysis Based on Absolute Isometric Contraction Torque

**Figure 6:**
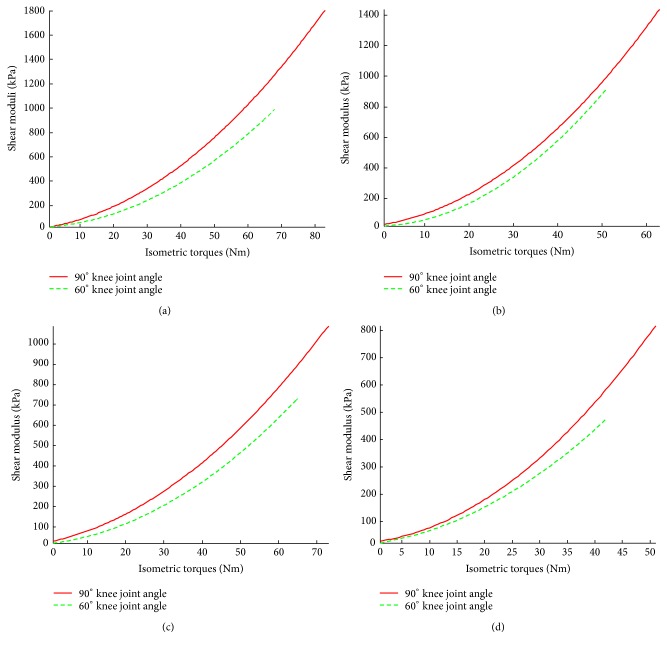
Comparison of the relationships between VI shear modulus and absolute torque at 90° and 60° knee joint angles for (a) young male subjects; (b) young female subjects; (c) elderly male subjects; and (d) elderly female subjects.

**Figure 7:**
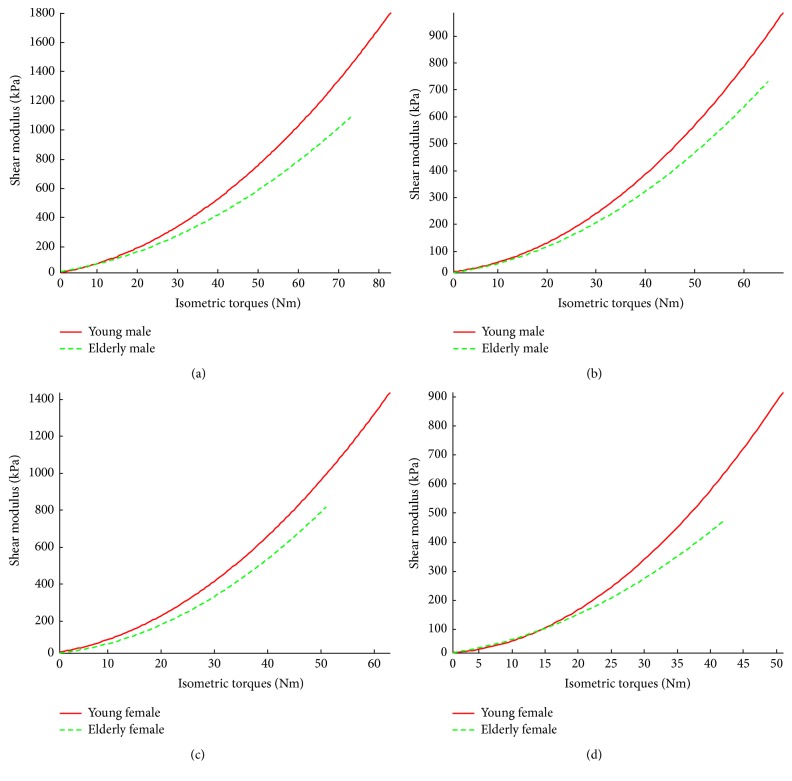
Age dependencies of the relationships between VI shear modulus and absolute torque. Comparisons were made between young and elderly male subjects at (a) 90° and (b) 60° knee joint angles and between young and elderly female subjects at (c) 90° and (d) 60° knee joint angles.

**Figure 8:**
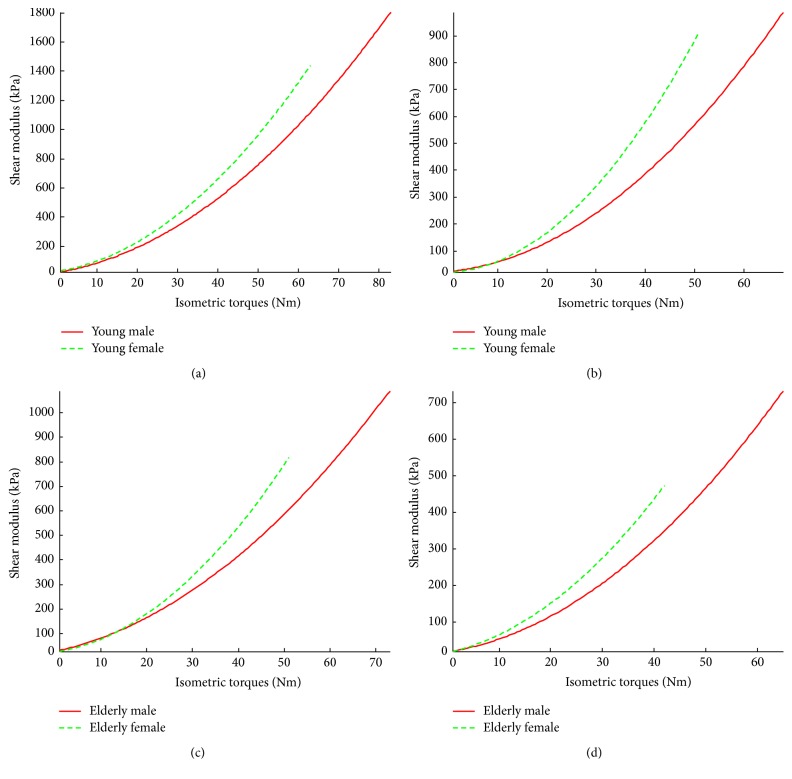
Gender dependencies of the relationships between VI shear modulus and absolute torque. Comparisons were made between young male and female subjects at (a) 90° and (b) 60° knee joint angles and between elderly male and female subjects at (c) 90° and (d) 60° knee joint angles.

Since, for each individual subject, the *R*^2^ value did not change when only the scale of the *x*-axis changed from the relative % MVC level to the absolute contraction torque, the quadratic regression model could also be used for examining the trend of the relationship between the VI shear modulus and absolute torque. The quadratic curves of each group were plotted to investigate the different trends at 90° and 60° knee joint angles, respectively. It was observed that, with the increasing absolute torque, VI shear modulus increased faster at a 90° knee joint angle than at a 60° knee joint angle, as demonstrated in Figure 6. This result was similar to the relationship between the VI shear modulus and relative muscle contraction level. The relationships between the VI shear modulus and absolute torque with different age ranges and the same sex at the same knee joint angle were also plotted (Figure 7). The results indicated that the VI shear modulus of young subjects increased faster compared with that of elderly subjects. This relationship also coincided with that between the VI shear modulus and relative muscle contraction level. Furthermore, the relationships between the VI shear modulus and the absolute torque of different sexes and the same age range at the same knee joint angle were also compared, as shown in Figure 8. The results showed that the VI shear modulus of females increased faster than that of males. However, this result was opposite to the relationship between the VI shear modulus and relative muscle contraction level. The reason for this conflict might be due to the relatively smaller MVC torque of the female subjects and will be further discussed in the following section.

## 4. Discussion

The current study provided some quantitative information regarding the VI stiffness at various contraction levels, especially the difference related to different age, sex, and joint angle.

### 4.1. Stiffness of VI in a Relaxed State

It was found in our study that the mean shear moduli of VI in a relaxed state for all subjects were 16.2 ± 8.1 kPa (at 90° knee joint angle) and 11.1 ± 4.4 kPa (at 60° knee joint angle). Although several methods based on shear wave velocity measurement have been used to estimate the shear modulus of muscle, few studies have been reported to assess the VI stiffness. The MRE method has been used on the quadriceps femoris muscles, but only on VL and VM. Bensamoun et al. [23] measured the shear modulus of VL and VM in a relaxed state on young healthy subjects. The mean values were 3.73 ± 0.85 kPa and 3.91 ± 1.15 kPa, respectively. The stiffness of VM was also assessed in another study [22], using both 2D MRE and 1D MRE methods, and the reported shear moduli were 4.36 ± 0.98 kPa and 3.69 ± 0.80 kPa, respectively. Although it was difficult to directly compare these results with ours since the target muscles were different, all of the measured shear modulus values fell into a similar range. It seemed that the VI muscle, which is a bipennate muscle, exhibited higher stiffness than VL and VM, in which the fiber orientation is unipennate. This difference may indicate that the propagation of shear waves is influenced by the muscle structure, such as muscle fiber orientation [23]. In addition, the different stiffness might be also related to muscle volume, muscle fiber type, muscle function, or other specific muscle characteristics. Furthermore, the results measured in a relaxed state might be also influenced by some other factors, such as the slight unconscious muscle tension during measurements and the momentary stiffness change due to the history of muscle use [29]. The effects of sports and exercises on muscle stiffness should be investigated in future studies. It was found that the mean value of VI shear modulus in a relaxed state measured at a 90° knee joint angle (16.2 ± 8.1 kPa) was significantly larger than that measured at a 60° knee joint angle (11.1 ± 4.4 kPa) (*p* < 0.001). Few studies have been performed to investigate the relationship between the VI shear modulus and knee joint angle. The shear moduli of relaxed tibialis anterior (TA) and lateral gastrocnemius (LG), which are muscles in distal leg, were studied using MRE at different ankle joint angles [34]. Shear modulus of LG increased to 35.1 ± 0.4 kPa at 20° of dorsiflexion from 22.1 ± 0.2 kPa at neutral but decreased to 18.4 ± 0.1 kPa at 45° of plantar-flexion. The shear modulus of TA increased from 12.3 ± 0.5 kPa at neutral to 32.5 ± 0.2 kPa at 45° of plantar-flexion and was almost unchanged at 20° of dorsiflexion (13.5 ± 0.4 kPa). These results indicated that muscle stiffness increased with the larger passive stretching. This was in agreement with our results. At a 90° knee joint angle, the VI was more stretched than at a 60° knee joint angle; thus it was reasonable that the shear modulus measured at 90° was larger. In addition to passive stretching, the difference of VI shear modulus might also be related to the morphology change with the different knee joint angles, such as fascicle length and pennation angle. When the knee was extended, the fascicle length of quadriceps femoris was reported to be shortened, and the pennation angle was reported to increase in step with the changes of knee joint angle [14, 15]. However, the detailed relationship between the changes of the VI stiffness and its morphology changes with the knee joint angle should be further studied.

### 4.2. Relationship between Muscle Stiffness and Step Isometric Contraction

As mentioned above, two methods have been used to exhibit the different contraction intensities, one is to use the relative muscle contraction level (% MVC), and the other one is to use the absolute torque of the knee joint extensor. In addition to these two methods, some previous studies also used different weight loads to represent the different muscle contraction levels, such as on the knee extensor [17] and on the elbow flexor [24]. However, the method using the weight load as an indicator was not accurate enough, since the length of limbs was different among the subjects and the lever of force was not counted.

#### 4.2.1. Comparison with Previous Studies Based on Relative Isometric Contraction Levels

Our results demonstrated that the VI stiffness along the muscle action direction was positively correlated to the relative muscle contraction level (% MVC) at two different knee joint angles and over the entire range of isometric contraction. Some previous studies have been reported about the positive correlation between the muscle stiffness and the nonfatigue muscle contraction level on different muscles. In the study of Bensamoun et al. [23], the reported shear moduli of VL and VM were 6.11 ± 1.15 kPa and 4.83 ± 1.68 kPa at 10% MVC isometric contraction level measured using the MRE method, and when at 20% MVC contraction, the shear moduli of VL and VM were reported to be 8.49 ± 4.02 kPa and 6.40 ± 1.79 kPa, respectively. They also reported that the shear moduli of VL and VM in a relaxed state were 3.73 ± 0.85 kPa and 3.91 ± 1.15 kPa. Their results showed a significant increase (*p* < 0.05) in the shear modulus of VL and VM with the increase in the muscle contraction level. Since the subjects in this stduy were young males and young females, but were not distinguished by sex, the different structure of VI from VL and VM, as we mentioned above, and the different posture and knee joint angle they used were difficult to use to directly compare the stiffness values they measured with ours. However, our conclusion that there was a significant increase in the VI shear modulus with the increase in the isometric contraction level from 10% to 20% MVC was in good agreement with their finding.

#### 4.2.2. Comparison with Previous Studies Based on Absolute Torques

In this study, it was demonstrated that the VI shear modulus along the direction of muscle action was also positively correlated to the absolute torque. Some previous studies have reported the shear modulus measured under different absolute torques of joint extensors or flexors, but the investigation was rarely focused on the quadriceps femoris muscles. Several studies have been reported to assess the stiffness of distal leg muscles, such as TA, LG, medial gastrocnemius muscle (MG), and soleus muscle (SL), as groups of agonist and antagonist muscles. Muscle shear modulus was measured under isometric contraction when the subject resisted ankle plantar-flexing and dorsiflexing moments, of which the directions of applied moments were opposite. In the study of Heers et al. [35] using the MRE method, the shear modulus of TA increased significantly (*p* < 0.0001) from 70.6 ± 1.8 kPa to 126.6 ± 5.1 kPa as the plantar-flexing moment increased from 8.2 N·m to 16.4 N·m. On the other hand, the shear modulus measured in the posterior muscles, that is, MG, LG, and SL, did not show significant changes (*p* > 0.05). Furthermore, when muscles resisted the dorsiflexing moments, as loads increased from 20.2 N·m to 40.4 N·m, the shear modulus increased significantly from 41.6 ± 5.1 kPa to 63.2 ± 5.1 kPa for MG (*p* < 0.01), from 27.6 ± 1.1 kPa to 73.1 ± 7.3 kPa for LG (*p* < 0.003), and from 36.0 ± 0.4 kPa to 65.6 ± 1.8 kPa for SL (*p* < 0.001). However, the shear modulus measured from TA did not change significantly with load (*p* > 0.05), and a similar study was performed by Jenkyn et al. [34] on the distal leg muscles. The shear moduli of relaxed TA and LG with the foot in a neutral position were 12.4 ± 0.5 kPa and 22.1 ± 0.2 kPa, respectively. Shear modulus of TA increased to 133.7 ± 2.1 kPa during a 16 N·m dorsiflexing effort but was relatively unchanged during a 48 N·m plantar-flexing effort (30.1 ± 0.5 kPa). Shear modulus of LG decreased slightly during a 16 N·m dorsiflexing effort (15.0 ± 0.3 kPa) but significantly increased with a 48 N·m plantar-flexing effort (140.4 ± 0.3 kPa). Since the muscles they studied were different from ours, their results could not be directly compared with ours [28]. However, our conclusion about the relationship between the increasing muscle stiffness and the increasing absolute torque was in good agreement with theirs.

#### 4.2.3. Regression Analysis of the Relationship between Muscle Stiffness and Activity

Our results showed that, over the entire range of isometric contraction, the stiffness of VI along the direction of muscle action was positively correlated to both the relative contraction level (% MVC) and the absolute torques (N·m). These two relationships were both close to a quadratic curve. In previous studies, the results of regression analysis were mostly reported as linear relationship [17, 24]. However, in these studies, the muscle shear modulus was only measured at 3–5 isometric contraction levels within a relatively smaller measurement range limited by the methods they used. In addition, it was a widely known approximation that a small range on the quadratic curve could be treated as being linear. That might be the reason of the difference between their conclusions and ours. Furthermore, from the results reported by Nordez and Hug [20], which measured the shear modulus of biceps brachii in a relatively larger muscle contraction range, the relationship between biceps brachii stiffness and isometric contraction torque seemed to be similar to a quadratic curve. However, they did not perform a quantitative regression analysis on their results in that paper.

#### 4.2.4. Comparison with Previous Studies Based on Different Joint Angles

In our study, the results of the mean MVC torque measured at different knee joint angles showed that the MVC torque measured at a 90° knee joint angle was larger than that measured at 60° (*p* = 0.001). This result was in agreement with previous studies [12, 13, 36, 37]. We also found that the shear modulus of VI measured at a 90° knee joint angle was larger than that measured at 60° during step isometric contraction (*p* < 0.001). Furthermore, for the two factors, interaction of joint angles and % MVC, with the increase of % MVC, and differences between the VI shear modulus measured at a 90° knee joint angle and those measured at a 60° also increased. Few studies have been reported to investigate the relationship between VI shear modulus and knee joint angle, especially under different step isometric contraction levels. Sasaki et al. [28] reported that, with the increase in the ankle joint angle, the shear modulus of TA measured by SSI also increases when performing submaximal voluntary contractions. This result is in agreement with ours. Additionally, EMG activity of VL muscle has been studied with different knee joint angles and the results can be used as a reference. Suter and Herzog [38] studied the EMG activity of the VL muscle at 15°, 30°, 45°, 60°, and 90° knee joint angles. They reported that, at a 90° knee joint angle, the EMG activity in MVC state was significantly higher than those at the other knee joint angles. It has also been indicated that, at a joint angle where the EMG activity was relatively larger, the corresponding MVC torque was also larger [12, 13]. Considering the highly positive correlation between muscle stiffness and isometric contraction torque observed in this study, our results about the VI stiffness at different knee joint angles were considered to be reasonable. Furthermore, similar to what was discussed in a relaxed state, the larger muscle stiffness at a 90° knee joint angle might also be related to the relatively larger passive stretching on VI, and the morphological differences at different knee joint angles might also contribute to the different VI stiffness. However, all of these hypotheses should be further investigated in future studies.

### 4.3. Age Effect on the Relationship between Muscle Stiffness and Step Isometric Contraction

When performing step isometric contraction, the effect of age factor became significant. The VI shear modulus of young subjects was larger than that of elderly. The results regarding age differences have been also reported by several previous studies [5, 39]. However, our results further showed that, in a relaxed state with either 60° or 90° knee joint angles, there was no significant effect of age found. The mechanism behind this remains unclear.

On the other hand, by comparing the trends of VI shear modulus and absolute torque with different age ranges and the same sex, it was shown that the VI shear modulus of young subjects increased faster than that of elderly subjects at the same knee joint angle. This was coincident with the age effect on the relationship between VI shear modulus and relative muscle contraction level (% MVC).

Previous studies on the age differences of muscle shear modulus in a relaxed state and under step isometric contraction conditions were even rarer than those on the sex differences. Domire et al. [27] measured the stiffness of TA using MRE in a relaxed state on 20 female subjects with an age range of 50–70 years. They found that there was no significant effect of age on muscle stiffness. This is in agreement with our results. Musculotendinous and musculoarticular stiffness have been also widely studied for the age difference. In the study of Ochala et al. [39], the stiffness of plantar flexor muscles was measured at 20%, 40%, 60%, and 80% MVC levels on young and elderly subjects. From the figures they published, it was clearly shown that the musculotendinous stiffness of elderly subjects was smaller than that of young subjects when measured at the same contraction level. This result of reduced musculotendinous stiffness due to muscle ageing is a valuable reference for our study. The age differences of morphology factors are related to the sarcopenia caused by the muscle ageing process and have been studied by many previous studies [6–8, 40]. However, few studies had been performed to evaluate the relationship between these morphology changes with muscle ageing and the muscle stiffness. Larsson et al. [41] found that the relative percentage of type I fibers increased with ageing and the fiber atrophy was most pronounced for type II fibers. As mentioned above, type I fibers were stiffer than type II fibers when generating equal active force. Therefore, the increasing percentage of type I fibers in elderly subjects should make the muscle stiffer in comparison with the young people when performing the same absolute contraction torque. However, this was opposite to the results observed in our study. One possible reason may be related to the increasing muscle coactivation level for the elderly [39, 40]. In our experiments, elderly subjects were also observed to be more strenuous when generating and keeping the isometric contraction levels. They noticeably tried to inspire the muscles in their whole body to accomplish the tasks. These coactivated muscles might share the original responsibility of VI and make the force generated by VI reduced comparing to the younger people. It is believed that the relative percentage of fiber types might not be the only or dominant factor for VI stiffness.

### 4.4. Sex Effect on the Relationship between Muscle Stiffness and Step Isometric Contraction

The mean MVC torque of knee extenders obtained from different sexes showed that the MVC torque of males were larger than that of females (*p* < 0.001). This result was in agreement with many previous studies [11, 29]. It was found that at both knee joint angles there was no significant main effect of sex (*p* > 0.5) on the VI shear modulus in a relaxed state. However, when performing step isometric contraction, the effect of sex factor was significant. The VI shear modulus of males was larger than that of females (*p* < 0.001). On the other hand, by comparing the trends of the relationship between VI shear modulus and absolute torque of the two sexes, it was found that the VI shear modulus of females increased faster than that of males under the same knee joint angle. This was opposite to the sex effect on the relationship between the VI shear modulus and relative contraction level (% MVC).

The sex effect on the shear modulus of skeletal muscles in a relaxed state and under step isometric contraction conditions has been rarely studied using methods based on shear wave velocity measurement, such as MRE or SSI. Van De Steeg et al. [29] measured the stiffness of four different muscles (biceps brachii, flexor digitorum profundus, soleus, and gastrocnemius) in a relaxed state in eight young male and four female subjects using MRE. They reported that the comparisons between females and males did not reveal statistically significant differences for any muscles. Our results agreed well with theirs. Although few previous studies which were related to the sex difference of muscle shear modulus during isometric contraction could be found, some results using quick-release movement and sinusoidal perturbations methods to evaluate the musculotendinous stiffness and musculoarticular stiffness may be used as references. Ochala et al. [39] found that the musculotendinous and musculoarticular stiffness were both higher in elderly females than those in elderly males at the same absolute contraction torque. This result was coincident with ours. Granata et al. [42] found that, at 0% and 20% MVC levels, the musculoarticular stiffness of quadriceps femoris muscles and hamstring muscle in young males was larger than that in young females. This result also echoed with our findings. Sex differences on morphology parameters may be another reason for the different muscle stiffness between males and females when performing isometric contraction [9–11]. However, these geometric factors would result in a complex relationship with muscle stiffness and the detailed influence of morphology parameters is still unclear. Another possible reason may be the sex difference of fiber type distribution, as we know that the slow-twitch (type I) and fast-twitch (type II) fibers have different stiffness characteristics. For instance, in rats, type I fibers had been observed to be stiffer than type II fibers when generating the same active contraction force [36, 37]. Therefore, the relatively greater area occupied by type I fibers in females and the greater area occupied by type II fibers in males [11, 43] can at least partially explain the higher muscle stiffness in females than in males at the same absolute contraction torque. Interestingly, it has been reported that the incident rate of fall is higher among females than males and more in elderly than young population. Though muscle stiffness is not currently regarded as a fall risk indication, it will be our future research topic following the study.

## 5. Conclusions

In this study, the relationship between VI stiffness and relative isometric contraction level or absolute torque generated by the knee extensor were studied over the entire muscle contraction range, that is, from 0% to 100% MVC. Meanwhile, the age and sex effects on the relationships were also systematically investigated. When performing isometric contraction, the VI stiffness of males was found to be significantly higher than that of females, and that of the young was higher than the elderly. Additionally, the stiffness measured at a 90° knee joint angle was always significantly larger than that measured at 60°. These results extended our knowledge on how age and sex can affect the muscle stiffness especially when the contractions are approximating the higher MVC and, therefore, may provide new insights into the age and sex bias in musculoskeletal studies, such as those on fall risks.

## Figures and Tables

**Table 1:** Demographic information of the subjects presented with mean (SD).

	Age (yr)	Height (cm)	Mass (kg)
Young males (*n* = 10)	29.4 (4.8)	174.7 (8.1)	73.3 (13.0)
Young females (*n* = 10)	27.6 (5.0)	164.3 (4.4)	55.3 (4.0)
Elderly males (*n* = 10)	60.6 (7.6)	166.7 (5.8)	66.1 (11.9)
Elderly females (*n* = 10)	56.7 (4.9)	156.9 (5.6)	58.9 (8.4)

**Table 2:** The mean MVC torque (N·m) of knee extensors in each group at 90° and 60° knee joint angles.

Knee joint angle		Young male	Young female	Elderly male
90°	166 ± 32.8	126 ± 34.0	146 ± 29.8	102 ± 30.4
60°	136 ± 18.4	102 ± 32.0	130 ± 28.6	84 ± 18.4

**Table 3:** The mean VI shear modulus (kPa) of each group in a relaxed state (0% MVC), shown separately for 90° and 60° knee joint angles.

Knee joint angle	Young male	Young female	Elderly male	Elderly female
90°	14.5 ± 6.3	19.0 ± 10.2	13.8 ± 7.4	17.2 ± 8.4
60°	11.6 ± 3.5	12.8 ± 5.4	10.3 ± 5.0	9.5 ± 3.3

**Table 4:** Multiway ANOVA showed significant main effects of percentage of MVC level on VI shear modulus. The post hoc Bonferroni comparisons results showed the details of the relationships among these levels.

Percentage of MVC	Post hoc comparisons results
0%	<30%–100%, *p* < 0.05
10%	<40%–100%, *p* < 0.005
20%	<50%–100%, *p* < 0.001
30%	>0%, *p* < 0.05; <50%–100%, *p* < 0.05
40%	>0%–10%, *p* < 0.005; <60%–100%, *p* < 0.001
50%	>0%–30%, *p* < 0.05; <70%–100%, *p* < 0.001
60%	>0%–40%, *p* < 0.001; <80%–100%, *p* < 0.001
70%	>0%–50%, *p* < 0.001; <80%–100%, *p* < 0.05
80%	>0%–70%, *p* < 0.05; <90%–100%, *p* < 0.005
90%	>0%–80%, *p* < 0.005; <100%, *p* < 0.001
100%	>0%–90%, *p* < 0.001

**Table 5:** Pearson's correlation coefficients between VI shear modulus and relative isometric contraction levels in each group and at different knee joint angles.

Knee joint angle	Young male	Young female	Elderly male	Elderly female
90°	0.977	0.974	0.980	0.975
60°	0.971	0.968	0.975	0.979
